# Potassium channel clustering: mechanisms shaping axonal excitability

**DOI:** 10.3389/fncel.2025.1627517

**Published:** 2025-07-01

**Authors:** Gabriel Escobedo, Matthew N. Rasband

**Affiliations:** Department of Neuroscience, Baylor College of Medicine, Houston, TX, United States

**Keywords:** axon, axon initial segment, node of Ranvier, scaffold, ion channel

## Abstract

The precise clustering of ion channels at axon initial segments (AIS) and nodes of Ranvier is essential for axonal excitability and rapid action potential propagation. Among the axonal ion channels, voltage-gated potassium channels (Kv) and two-pore domain potassium (K2P) leak channels are key regulators of AIS and nodal excitability. Kv7 and Kv1 channels contribute to action potential threshold and repolarization at the AIS, and membrane repolarization in axons has historically been attributed to Kv channels. However, recent studies suggest that at nodes of Ranvier K2P channels, particularly TRAAK and TREK-1, play a dominant role in action potential repolarization. The interaction of Kv and K2P channels with diverse scaffolding proteins ensures their precise localization at AIS and nodes. Mislocalization or dysfunction of axonal Kv and K2P channels can cause epilepsy and neurodevelopmental disorders. This review explores the diversity of potassium channels and the mechanisms responsible for their clustering at AIS and nodes of Ranvier. Understanding these processes will be essential for therapeutic strategies aimed at treating diseases characterized by abnormal potassium channel expression, clustering, and function in neurons.

## Introduction

The nervous system’s ability to efficiently and rapidly transmit electrical signals depends on the precise organization of excitable domains along myelinated axons, especially the axon initial segment (AIS) and nodes of Ranvier. The AIS serves as the primary site of action potential initiation, and functions to filter and integrate synaptic inputs before a neuronal response is generated. Nodes of Ranvier regenerate action potentials, thereby ensuring rapid conduction through saltatory propagation along myelinated axons (Huxley and Stampfli, 1949; Cohen et al., 2020). Together, myelination and saltatory conduction increase action potential conduction velocity while decreasing both the energy and space required (Ritchie, 1984).

The AIS and nodes of Ranvier feature high densities of voltage-gated sodium (Nav) channels, voltage-gated potassium (Kv) channels, and more recently were recognized to also be enriched with two pore-domain potassium (K2P) leak channels (Escobedo et al., 2024; Luque-Fernandez et al., 2024). The structural integrity and molecular organization of the AIS and nodes are maintained by the scaffolding protein AnkyrinG (AnkG) and a periodic actin-spectrin cytoskeleton. This specialized cytoskeleton maintains clustered ion channels and neuronal polarity (Hedstrom et al., 2008; Sobotzik et al., 2009; Liu et al., 2020b). Additionally, the AIS is plastic, undergoing structural and molecular modifications in response to neuronal activity, synaptic input, and injury, allowing neurons to modulate their excitability (Grubb and Burrone, 2010; Kuba et al., 2010; Jamann et al., 2021; Freal et al., 2023).

Historically, membrane repolarization in axons was attributed primarily to Kv channels (Hodgkin and Huxley, 1952b,a). However, in mammalian myelinated axons, membrane repolarization at nodes was later found to occur primarily through leak K^+^ channels (Chiu et al., 1979). Only recently were the K2P leak channels TRAAK and TREK-1 discovered to be the principal mediators of nodal repolarization (Brohawn et al., 2019; Kanda et al., 2019). Here, we discuss the diverse types of potassium channels found at the AIS and nodes of Ranvier, and the molecular mechanisms underlying their localization and function.

## Structure and function of the axon initial segment and nodes of Ranvier

**FIGURE 1:**
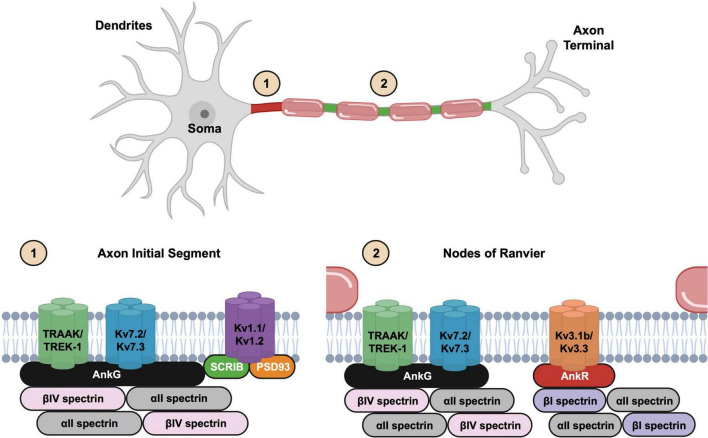
Illustration of neuronal structure and the molecular organization of the excitable domains. The axon initial segment (1) is located at the proximal axon while nodes of Ranvier (2) are found at gaps in the myelin sheath. Kv2.1 channels are not shown in the axon initial segments (AIS) cartoon.

The axon initial segment (AIS) is defined molecularly by the clustering of the scaffolding protein AnkG, while functionally it is the site of axonal spike initiation. In many neurons its anatomical location coincides with the axon hillock (Palay et al., 1968). However, some neurons have an axon that arises from a primary dendrite; these are called axon carrying dendrite (AcD) neurons. AcD neurons have their AIS at the proximal axon where it arises from the dendrite (Han et al., 2025). The AIS plays a critical role in the initiation and modulation of action potentials, and its structural features are tailored to support this function. The AIS functions as the “spike initiation zone” due to its high concentration of sodium and potassium channels (Figure 1). Together, these channels are responsible for action potential initiation, modulation, and propagation [see (Jenkins and Bender, 2024)].

Nodes of Ranvier are periodically spaced gaps in the myelin sheath along myelinated axons that like the AIS are highly enriched with many of the same ion channels (Figure 1). These small yet essential domains serve as the primary sites for action potential regeneration during saltatory conduction. The molecular architecture of nodes of Ranvier is like the AIS, and both include a variety of membrane and extracellular matrix molecules. These membrane and matrix proteins interact indirectly with AIS and nodal ion channels to promote their clustering and stability (Susuki et al., 2013; Desmazieres et al., 2014). The striking similarity in the molecular composition of the AIS and nodes is consistent with the notion that nodes are evolutionary derivatives of the AIS (Hill et al., 2008).

The AIS and nodal enrichment of ion channels, membrane proteins, and cell surface proteins depends on the master organizer and scaffolding protein AnkG. In fact, most AIS and nodal proteins bind directly to AnkG. There are three ankyrin scaffolding proteins in mammals: AnkyrinR (AnkR), AnkyrinB (AnkB), and AnkyrinG (AnkG), corresponding to the genes *ANK1–3*, respectively (Lux et al., 1990; Chan et al., 1993; Kordeli et al., 1995). Ankyrins include 24 ANK repeats in their membrane-binding domain that allow them to interact with diverse membrane and membrane-associated proteins (Sedgwick and Smerdon, 1999; Bennett and Healy, 2008). Among the ankyrins, only AnkG is enriched at the AIS, and it has been studied extensively for its role in clustering AIS proteins. AnkG was first shown to be essential for AIS Nav channel clustering (Zhou et al., 1998), and was later shown to be indispensable for clustering other AIS proteins (Zhou et al., 1998; Jenkins and Bennett, 2001; Hedstrom et al., 2007) and maintenance of axon identity and neuronal polarity (Hedstrom et al., 2008; Sobotzik et al., 2009). Both AnkG and AnkR can participate in ion channel clustering at nodes of Ranvier (Ho et al., 2014), and their loss completely abolishes nodal Nav and Kv channel clustering. Moreover, all three ankyrins can cluster Nav channels at the neuromuscular junction (Zhang et al., 2021).

Axon initial segment and nodal AnkG is linked to actin through a βIV/αII spectrin tetramer (Berghs et al., 2000; Yang et al., 2007; Huang et al., 2017). Electron microscopy shows this AnkG-spectrin-actin protein complex appears beneath the plasma membrane as an electron-dense undercoat (Palay et al., 1968; Vassilopoulos et al., 2019). Newer single molecule localization microscopy imaging methods revealed these cytoskeletal proteins have a periodic arrangement with rings of actin spaced at ∼190 nm intervals by spectrin tetramers that are linked to the plasma membrane through AnkG (Xu et al., 2013; Leterrier et al., 2015).

AnkyrinG clustering at the AIS is cell autonomous. In contrast, clustering of AnkG at nodes of Ranvier requires the help of myelinating glia. During early development, a distal axonal cytoskeleton consisting of AnkB, βII/αII spectrin assembles first and creates an intra-axonal boundary restricting AnkG and βIV/αII spectrin to the proximal axon. Thus, the distal end of the AIS functions as an intra-axonal boundary restricting AnkG to the proximal axon (Galiano et al., 2012).

AnkyrinG functions not only to link membrane proteins to the cytoskeleton, but it may also stabilize AIS and nodal ion channels indirectly through its interaction with the extracellular matrix (Hedstrom et al., 2007). Thus, AnkG functions as a hub to connect ion channels to both intracellular cytoskeletons and the extracellular matrix “exoskeleton.” This may account for the extreme stability and resistance of many AIS proteins to detergent solubilization (Garrido et al., 2003; Boiko et al., 2007). The nodal extracellular matrix also forms the basis for node of Ranvier assembly in the peripheral nervous system (PNS). At PNS nodes of Ranvier, AnkG interacts with the PNS node-specific extracellular matrix protein gliomedin through the cell adhesion molecule NF186 (Eshed et al., 2005). During early developmental myelination, the clustering of nodal ion channels begins at heminodes located at the edges of myelinating Schwann cells (Vabnick et al., 1996). Schwann cells express secreted gliomedin and NrCAM that cluster NF186 at heminodes on the axonal membrane (Eshed et al., 2005). NF186 functions as an attachment site for AnkG, and AnkG recruits Nav and Kv channels. The entire NF186-AnkG-ion channel complex is stabilized through binding to βIV/αII spectrin within the periodic cytoskeleton. Besides the gliomedin-dependent mechanism in the PNS, the paranodal junctions, formed between the myelin sheath and axon flanking nodes of Ranvier, comprise a second mechanism of nodal ion channel clustering. Paranodal cell adhesion molecules recruit βII/αII spectrin and this specialized cytoskeleton functions to prevent the lateral diffusion of ion channels, thereby restricting their location to nodes of Ranvier (Zhang et al., 2013; Amor et al., 2017). In the central nervous system (CNS), the formation of the paranodal βII/αII spectrin-dependent boundary is the main mechanism for the developmental clustering of nodal ion channels (Susuki et al., 2013; Rasband and Peles, 2021).

The AIS also maintains neuronal polarity and axon identity, but the mechanisms remain incompletely understood; loss of AnkG allows proteins normally restricted to the somatodendritic compartment to enter axons. Thus, for some proteins the AIS functions as a barrier, and this barrier exists both at the level of the membrane and transport (Nakada et al., 2003; Song et al., 2009). In contrast, loss of AnkG also causes a reduction in the density of axonal RNAs (Teliska et al., 2022), suggesting AIS structures may also facilitate the entry of some proteins, cargoes, and organelles. Possibly related to polarity, one hallmark of the AIS is its dense, bundled microtubules, cross-linked by proteins such as TRIM46. Some studies suggest these bundled microtubules are important for polarized trafficking (van Beuningen et al., 2015; Harterink et al., 2019). However, recent experiments in TRIM46-deficient mice show neuronal polarity is intact in the absence of fasciculated microtubules (Melton et al., 2024). Thus, the function of the bundled microtubules found at the AIS remains obscure.

## Potassium channel clustering and localization in axons

With this background on AIS and nodes of Ranvier in mind, we turn our focus to the mechanisms responsible for potassium channel clustering at these axonal excitable domains. Kv7, Kv1, Kv2, Kv3, and K2P channels each use distinct mechanisms, scaffolding proteins, and protein interactions to facilitate their clustering at excitable domains.

### Kv7 channels

**FIGURE 2:**
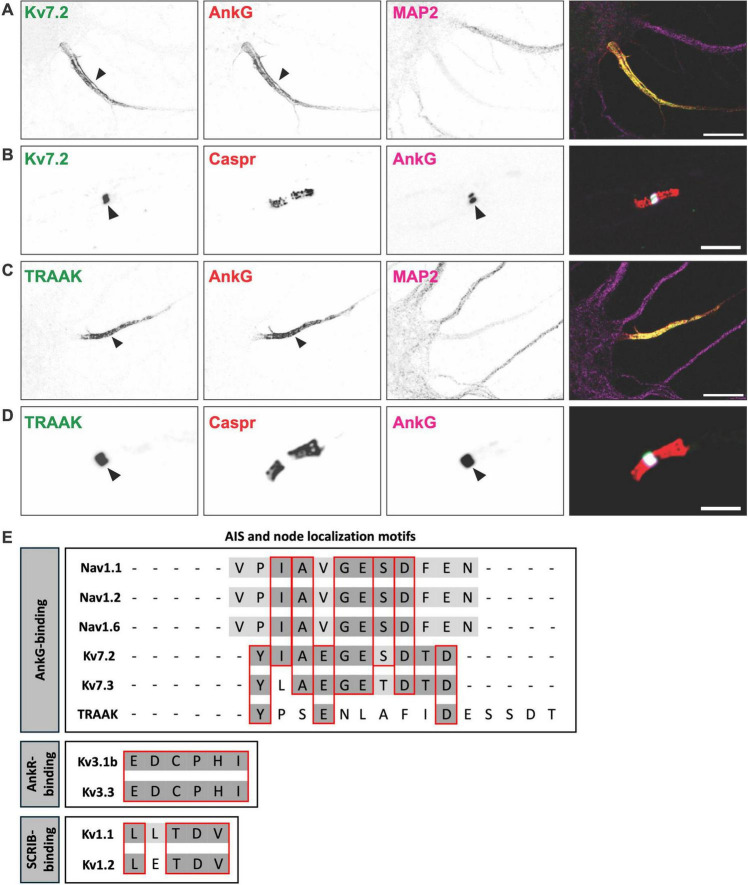
K^+^ channels found at axon initial segments and nodes of Ranvier. **(A)** Axon initial segment of a cultured hippocampal neuron immunostained for Kv7.2 (green), AnkyrinG (AnkG) (red), and MAP2 (magenta). Scale bar, 10 μm. **(B)** Node of Ranvier immunostained for Kv7.2 (green), Caspr (red), and AnkG (magenta). Scale bar, 10 μm. **(C)** Axon initial segment of a cultured hippocampal neuron immunostained for the K2P channel TRAAK (green), AnkG (red), and MAP2 (magenta). Scale bar, 10 μm. **(D)** Node of Ranvier immunostained for the K2P channel TRAAK (green), Caspr (red), and AnkG (magenta). Scale bar, 10 μm. **(E)** Axon initial segments (AIS) and node of Ranvier localization motifs for voltage-gated sodium (Nav), voltage-gated potassium (Kv), and two pore-domain potassium (K2P) channels.

Kv7 channels are heterotetramers comprised of Kv7.2 and Kv7.3 α-subunits. Kv7 channels generate the M-current, which stabilizes the resting membrane potential and prevents excessive neuronal firing (Shah et al., 2008). Kv7 channels were among the first potassium channels to be identified at AIS and nodes (Devaux et al., 2004; Figures 2A, B). At nodes, Kv7 channels underlie the slow nodal potassium current and stabilize membrane potential. As such, blocking nodal Kv7 channels leads to an increase in axonal excitability (Schwarz et al., 2006). Consistent with the notion that Kv7 channels are responsible for the slow nodal potassium current, patients with a point mutation in the Kv7.2 voltage sensor have myokymia (muscle rippling) and loss of frequency adaptation (Dedek et al., 2001).

Kv7 channel enrichment at the AIS and nodes is mediated by a conserved nine amino acid motif (IAEGES/TDTD) in their C-terminal region, that binds directly to AnkG (Figure 2E). This motif is homologous to the Nav channel AnkG-binding motif (Garrido et al., 2003; Lemaillet et al., 2003; Pan et al., 2006). Despite its high degree of similarity, the motif is thought to have evolved independently and represents a remarkable example of convergent molecular evolution (Hill et al., 2008). This motif is sufficient for its interaction with AnkG, and loss of AnkG disrupts Kv7 channel clustering at the AIS (Pan et al., 2006). As noted above, in the absence of nodal AnkG, Nav channel clustering may be rescued by nodal AnkR. However, loss of nodal AnkG is accompanied by a complete loss of nodal Kv7.2, suggesting that although its ankyrin-binding motif is homologous to Nav channels, AnkR cannot cluster Kv7 channels (Wang et al., 2018).

Kv7 channel interaction with AnkG, and presumably its localization at the AIS and nodes, is regulated by phosphorylation. Protein kinase CK2 phosphorylates the AnkG-binding motif of Kv7.2, enhancing its interaction with AnkG (Xu and Cooper, 2015). This phosphorylation dependent regulation suggests that Kv7.2 localization may be dynamic in response to neuronal activity or extracellular signals. Given that CK2 activity is known to be influenced by synaptic input and intracellular signaling, it is possible that Kv7 channel clustering at the AIS is plastic, allowing neurons to fine tune their excitability under different physiological conditions (Kimura and Matsuki, 2008). The identity of other kinases or the phosphatases that regulate the stability and function of Kv7 channels is unknown, but it is clear that phosphorylation is an additional layer of regulation.

### Kv1 channels

Heterotetrameric Kv1 channels consisting of Kv1.1, Kv1.2, and Kv1.4 α-subunits are enriched at the AIS (Lorincz and Nusser, 2008; Ogawa et al., 2008). While AIS Kv7 channels regulate subthreshold excitability, AIS Kv1 channels modulate high-frequency firing and the shape of the action potential waveform (Kole et al., 2007; Rama et al., 2017). Immunostaining of AIS Kv1 channels showed that they are more highly enriched in the distal regions of the AIS (Van Wart et al., 2007; Lorincz and Nusser, 2008). In addition, Kv1 channels are not found at nodes of Ranvier. Instead, they are excluded from nodal and paranodal domains and are clustered beneath the myelin sheath on each side of the node at the juxtaparanodal domain (Wang et al., 1993). Together, these observations suggest the mechanisms of AIS Kv1 channel clustering are unique and may rely on scaffolding proteins or mechanisms other than AnkG.

Kv1 channel α-subunits have a conserved C-terminal PDZ binding motif (L/ETDV) (Figure 2E) and can be clustered on the cell surface by PDZ domain-containing proteins of the membrane-associated guanylate kinase (MAGUK) family (Kim et al., 1995). Among the MAGUKs, the postsynaptic density 93 (PSD93) scaffolding protein was found to be enriched at the AIS (Ogawa et al., 2008). *In vitro* knockdown experiments demonstrated that reducing PSD93 levels also reduced AIS Kv1 channel clustering. However, subsequent studies in PSD93 knockout mice showed that Kv1 channels remained clustered at the AIS, indicating that PSD93 is not essential for AIS Kv1 channel clustering and that other scaffolding proteins must also contribute to their localization (Ogawa et al., 2010).

For more than a decade, these contradictory *in vitro* and *in vivo* results remained unresolved. Recently, our lab used immunoproximity biotinylation to perform a deep proteomic analysis of the AIS (Zhang et al., 2023). This was followed by CRISPR-mediated genome editing to endogenously tag all PDZ domain-containing proteins found in the AIS proteome. Among the 18 candidates examined, this knock-in strategy revealed two PDZ domain-containing scaffolding proteins enriched at the AIS: PSD93 and SCRIB. SCRIB was previously reported to be a regulator of dendrite development, spine morphology, and synaptic plasticity (Moreau et al., 2010; Szczurkowska et al., 2020). Additional experiments showed that SCRIB co-immunoprecipitates with Kv1 channels and co-expression of SCRIB with Kv1 channels is sufficient to induce their surface clustering with Kv1 channels. In contrast, loss of SCRIB significantly reduces Kv1 channel clustering at the AIS. AnkG is the master organizer of the AIS, and while loss of PSD93 reduces AIS Kv1 channels *in vitro*, loss of AnkG completely abolishes Kv1 channel clustering. So how is SCRIB connected to AnkG and PSD93? SCRIB can not only bind to and cluster Kv1 channels, but it can also recruit AnkG into those surface clusters and it co-immunoprecipitates with SCRIB. Loss of both SCRIB and PSD93 *in vitro* abolishes Kv1 channel clustering. Thus, SCRIB functions as a bridge between AnkG and Kv1 channels, and acts synergistically with PSD93 to cluster Kv1 channels (Zhang et al., 2025). Although AIS Kv1 channel clustering depends on AnkG, Kv1 channels do not interact directly with AnkG. Intriguingly, although it binds directly to AnkG, SCRIB is found neither at nodes nor juxtaparanodes (Zhang et al., 2025). The mechanisms that restrict SCRIB to the AIS and exclude them from distal axons are unknown. AIS localization of Kv1 channels may also rely on posttranslational modifications. For example, some evidence supports the idea that the palmitoyl acyltransferase ZDHHC14 palmitoylates both Kv1 channels and PSD93; loss of ZDHHC14 eliminates the AIS clustering of both Kv1 channels and PSD93 (Sanders et al., 2020).

### Kv2 channels

The delayed rectifier Kv2.1 channel, found primarily in somatodendritic domains of cortical and hippocampal neurons (Antonucci et al., 2001) may also be found at the AIS (Sarmiere et al., 2008). However, the physiological functions of AIS Kv2.1 remain poorly understood. In addition, Kv2.1 has not been reported at nodes of Ranvier, and unlike Kv7, Kv1, and K2P channels, it is not uniformly distributed along AIS, but rather it is found in clusters, at endoplasmic reticulum-plasma membrane (ER-PM) junctions that form in “holes” in the AnkG-dependent cytoskeleton. Unlike other Kv channels, AIS clustering of Kv2.1 channels does not occur through an Ankyrin-directed protein complex. Interestingly, the vesicle-associated membrane protein-associated proteins isoforms A and B (VAPA and VAPB) colocalize with and bind to Kv2.1, and loss of VAPA reduces the amount of Kv2.1 at ER-PM junctions. However, Kv2.1 appears to recruit VAPs to ER-PM junctions (Kirmiz et al., 2018). Nevertheless, Jensen et al. (2017) identified a 20 amino acid region (residues 720–745) in the C-terminus of Kv2.1 that is necessary for AIS localization. In addition, within this domain they found two phosphorylation sites (T728 and S732) that when mutated to alanine abolish Kv2.1’s AIS localization. Thus, like Kv7 and K2P channels (see below) the AIS localization of Kv2 channels depends on phosphorylation. Future studies should focus on the putative scaffolding proteins responsible for AIS Kv2.1 clustering at ER-PM junctions.

### Kv3 channels

The Kv3 subfamily of Kv channels functions mainly in neurons that engage in high-frequency firing. Among the Kv3 channels, Kv3.1b and Kv3.3 have been reported at subsets of mammalian nodes of Ranvier in the CNS, but not at the AIS (Devaux et al., 2003). In contrast, Kuba et al. (2015) reported Kv3.1-containing channels at the AIS in chick brainstem auditory nuclei. Unlike Kv7.2, Kv7.3 and K2P channels, which regulate resting membrane potential and subthreshold excitability, Kv3.1b and Kv3.3 are activated at more depolarized voltages, possibly contributing to the rapid repolarization of nodes (Hernandez-Pineda et al., 1999; Rudy and McBain, 2001). As described above, Nav, Kv7, and K2P channels are clustered at nodes by AnkG. However, Kv3.1b and Kv3.3 are not clustered by AnkG but instead rely on AnkR (Stevens et al., 2021; Stevens et al., 2022). The binding between AnkR, Kv3.1b, and Kv3.3 occurs through a conserved 6-amino acid AnkR-binding motif (EDCPHI) located in the C-terminus of the channel subunits (Figure 2E). Thus, nodes that have AnkG, but no AnkR, have clustered Kv7 and K2P channels. In contrast, nodes that have AnkR, but no AnkG, have only Kv3 channels (Stevens et al., 2021), and nodes with both AnkR and AnkG have Kv7, Kv3, and K2P channels. This remarkable diversity, complexity, and clustering of nodal potassium channels is dictated by the types of ankyrin scaffolding proteins found at mammalian nodes of Ranvier. The striking observation of Kv3.1 channels at the AIS of neurons in avian brainstem auditory nuclei suggests that avians may have evolved distinct Kv3.1 channel clustering mechanisms at the AIS (Kuba et al., 2015). In mammals, we never observed AIS AnkR (Liu et al., 2020a), which may explain the absence of AIS Kv3 channels.

### K2P channels

TWIK-related arachadonic acid activated K^+^ channel (TRAAK) and TREK-1 are closely related members of the K2P channel family that display both thermal and mechanical sensitivity (Maingret et al., 1999a; Maingret et al., 1999b). K2P channels are heterodimers, expressed at low levels in neurons, and underlie leak potassium currents (Talley et al., 2001). Unlike Kv channels that are gated by membrane potential changes, K2P channels provide voltage-independent background leak potassium currents, influencing neuronal excitability by stabilizing the resting membrane potential (Enyedi and Czirjak, 2010). Although a nodal leak potassium current was proposed decades ago (Chiu et al., 1979), only recently were TRAAK and TREK-1 identified as the molecular basis of the leak current at nodes of Ranvier (Brohawn et al., 2019; Kanda et al., 2019). The shift from Kv channel mediated repolarization in invertebrates and unmyelinated axons to K2P-mediated repolarization in mammals represents a critical evolutionary adaptation that optimizes conduction velocity (Tonomura and Gu, 2022).

Immunostaining experiments indicated that K2P channels were not found at the AIS, suggesting that they may be clustered through an AnkG-independent mechanism. However, subsequent studies using glyoxal as a fixative (Konno et al., 2023), rather than paraformaldehyde, revealed AIS TRAAK and TREK-1 (Escobedo et al., 2024). Thus, like Kv7 channels, K2P channels are found at both AIS and nodes of Ranvier (Figures 2C, D). Although the function of AIS K2P channels has not been investigated, it is reasonable to assume they are primary drivers of action potential repolarization as they are at nodes of Ranvier.

The AIS and nodal clustering of TRAAK and TREK-1 depends on AnkG. A structure-function analysis revealed that TRAAK has a highly conserved AnkG-binding motif in its C-terminal region (YPSENLAFIDESSDT), that is both necessary and sufficient for its clustering at the AIS (Escobedo et al., 2024; Luque-Fernandez et al., 2024). This sequence has little similarity to that of Nav and Kv7 channels (Figure 2E), suggesting that TRAAK’s AnkG-binding motif evolved independently. In support of the notion that AIS ion channel densities are plastic and can be dynamically modulated, TRAAK’s affinity for AnkG depends on phosphorylation, just like Kv7 and Nav channels. In contrast, TREK-1 does not have an AnkG-binding motif and does not independently localize to the AIS. Instead, TREK-1 clustering requires its co-assembly with TRAAK (Escobedo et al., 2024). Thus, heterodimerization between TRAAK and TREK-1 is required for TREK-1 localization at excitable domains such as the AIS. AnkG conditional knockout mice have normal Nav channel clustering due to compensation by AnkR. However, these AnkG-deficient nodes of Ranvier lack both Kv7 and K2P channels, indicating that the complement of nodal potassium channels has a high degree of specificity for their binding to ankyrin scaffolding proteins (Wang et al., 2018; Escobedo et al., 2024).

## Conclusion and future directions

Voltage-gated potassium channels and K2P channel dysfunction and mislocalization are linked to neurological diseases, including epilepsy, multiple sclerosis, and neurodevelopmental disorders (Browne et al., 1995; Biervert et al., 1998; Singh et al., 1998; Smart et al., 1998; Bauer et al., 2018; Djillani et al., 2019). The diverse types of potassium channels and their unique clustering mechanisms highlight the evolutionary pressure to optimize action potential conduction in myelinated axons. A deeper understanding of the mechanisms regulating these scaffolding proteins and their interactions with axonal potassium channels may offer new therapeutic strategies to restore channel localization and function.

Despite our understanding of the functions of axonal potassium channels and their mechanisms of clustering, important questions remain. For example, although the contributions of AIS Kv7 and Kv1 channels to action potential initiation are now well-understood (Kole et al., 2007; Shah et al., 2008; Rama et al., 2017), the role of AIS K2P channels has not been investigated. In addition, the kinases that phosphorylate TRAAK, and how this phosphorylation influences AIS and nodal properties remain unknown. In contrast to Nav channels and Neurofascin (Wang et al., 2014; He et al., 2022), the structural features of the ankyrin-binding motifs that confer specificity for each kind of ankyrin remain poorly understood. Finally, it is important to determine if and how AIS and nodal potassium channel clustering is dynamically regulated during development, plasticity, and following injury. Although it is widely accepted that the AIS can undergo structural modifications in response to neuronal activity, if and how these changes impact potassium channel clustering, or if the density or distribution of the potassium channels themselves change is unknown. Thus, much remains to be done to fully understand potassium channel regulation in axons, and to apply these discoveries to treat diseases characterized by abnormal potassium channel expression, clustering, and function in neurons.
